# Cluster Headache and the Comprehension Paradox

**DOI:** 10.1007/s42399-021-01083-z

**Published:** 2022-01-10

**Authors:** Heiko Pohl, Andreas R. Gantenbein, Peter S. Sandor, Jean Schoenen, Colette Andrée

**Affiliations:** 1 Department of Neurology, University Hospital Zurich, Frauenklinikstrasse 26, 8091 Zurich, Switzerland; 2 Zurzach Care, Bad Zurzach, Switzerland; 3 Headache Research Unit, Department of Neurology, Citadelle Hospital, University of Liège, Liège, Belgium; 4 Migraine Action Switzerland, Bottmingen, Switzerland; 5 Department of Pharmaceutical Sciences, University of Basel, Basel, Switzerland

**Keywords:** Burden of disease, Primary headache, Social interaction, Interictal burden

## Abstract

Patients with primary headache disorders such as cluster headache cycle between being entirely healthy and almost completely incapacitated. Sick leave or reduced performance due to headache attacks demands flexibility by their social counterparts. The objective of this study is to test the hypothesis that headache patients cause frustration that grows with the times colleagues have to take over their work. In this study, we analysed cluster headache patients’ answers to an online questionnaire. Participants self-reported their number of sick days, the number of days on which leisure activities were missed and whether they felt understood by colleagues and family. We then investigated the correlation between the number of sick days and the proportion of patients feeling understood by colleagues and friends. We found that feeling understood by colleagues and friends decreases with a growing number of sick days. However, when sick days accrue further, this proportion increases again. The number of sick days correlates similarly with both colleagues’ and friends’ understanding. The number of cluster headache patients feeling understood by others decreases with an increasing number of sick days. Their social circles’ frustration with the patients’ failure to meet obligations and expectations are a likely reason. With a growing number of sick days, however, the portion of patients feeling understood rises again despite patients meeting others’ expectations even less. This ‘comprehension paradox’ implies the influence of other factors. We suspect that growing numbers of sick days foster understanding as the disability of the disease becomes increasingly apparent.

## Introduction

Patients with primary headache disorders such as migraine or cluster headache (CH) cycle between being entirely healthy and almost completely incapacitated [12, 20]. Fluctuating performances jeopardise any planning and complicate meeting deadlines. The challenge of headache patients is to find ways of meeting personal or professional obligations despite the pain.

Many patients with CH or migraine report that friends and colleagues do not understand and accept the limitations imposed by the disorder [13, 17]. The reason for lacking comprehension might be that the headaches affect not only patients but also their co-workers and friends.

Patients’ sick leave or reduced performance due to headache attacks demands flexibility by their social counterparts [21]. Unfilled orders create additional work; cancelling appointments on short notice can create disappointment. Presumably, headache patients cause frustration that grows with the times colleagues have to take over their work.

This study aims to investigate if there is a correlation between the number of CH patients’ sick days and the proportion of patients feeling understood by others.

## Methods

This study is a secondary analysis of data collected by the EUROLIGHT Cluster Headache Project [3].

### Patients

We only included patients with a validated diagnosis of CH; please see our previous paper for further details on the validation method [3]. In brief, we validated the diagnosis based on the reported symptoms and excluded all patients whose self-reported symptoms did not meet the diagnostic criteria published in edition 3 beta of the International Headache Classification [10]. Patients enrolled from May to August 2012.

### Study Design

We invited patients in several European countries to anonymously complete an online questionnaire that consisted of a modified version of the EUROLIGHT questionnaire that we discussed in a different publication [3]. The available data determined the sample size.

Here, we will focus on the number of days employed patients had been unable to go to work (‘sick days’), as well as on the number of days patients had missed social activities (‘missed leisure days’). We assessed the former with the following question, ‘On how many days in the last 3 months could you not go to work or school because of your headaches?’; the latter was assessed asking, ‘On how many days in the last 3 months did you miss family, social or leisure activities because of your headaches?’.

In addition, we will include the answers to the following two questions into the analysis. (i) ‘Do you feel that your family and friends understand and accept your headaches?’ (ii) ‘Do you feel that your employer and work colleagues understand and accept your headaches?’ Participants could choose between the answers ‘yes’ and ‘no’.

Finally, we compared the feeling of being understood with the scores of the anxiety and depression subscales of the Hospital Anxiety and Depression Scale (HADS-A and HADS-D, respectively) [24]. As suggested by Hinz and Brähler, we considered scores of eight and more points in the subscales to indicate elevated levels of anxiety and depressive symptoms [11].

### Statistical Analysis

We subdivided the patients into subgroups according to the number of sick days and missed leisure days. Then, we calculated the proportion of patients who had reported feeling understood by colleagues and employers as well as family and friends, respectively, for each subset.

Proportions are reported as percentages, averages as means and standard deviations, and categorical variables as frequencies. The two-sided chi-squared test allowed testing for statistically significant differences between two subgroups; to quantify differences, we also calculated odds ratios (OR) and their 95% confidence intervals (95% CI). The Mann–Whitney *U* test allowed testing the influence of a dichotomous variable on an ordinal scaled variable. We performed the statistical analysis with SPSS version 25 and set the significance level at 0.05. Missing data is referred to as ‘not reported’ (n.r.).

## Results

We were able to validate the CH diagnosis in 1165 and took their data for further analysis. Of them, 412 patients were occupied and provided information on absenteeism during the last 3 months and comprehension of colleagues and employers; 279 were male (279/411, 67.9%; 1 n.r.), and their average age was 40 ± 10 years (3 n.r.). Episodic CH was reported by and validated in 299 (299/398, 72.6%, 14 n.r.) of whom 222 were in-bout (222/299, 74.2%); 99 participants had chronic CH (99/398, 24.9%; 14 n.r.).

Furthermore, we subdivided the patients into subgroups according to the number of sick days in the last 3 months. In that period, patients with episodic CH had 11 ± 17 (median: 4 days) sick days and patients with chronic CH had 23 ± 30 days (median: 8 days); all participants taken together were absent from work on 14 ± 22 days (median: 5 days). Most commonly, participants recalled between 0 and 10 days of absence from work (276/412, 67.0%). There were 54 patients with zero sick days, 222 with 1 to 10, 52 with 11 to 20, 30 with 21 to 30 and 31 with 31 to 60 sick days. Finally, there were 23 in the last group with more than 60 sick days.

**Fig. 1:**
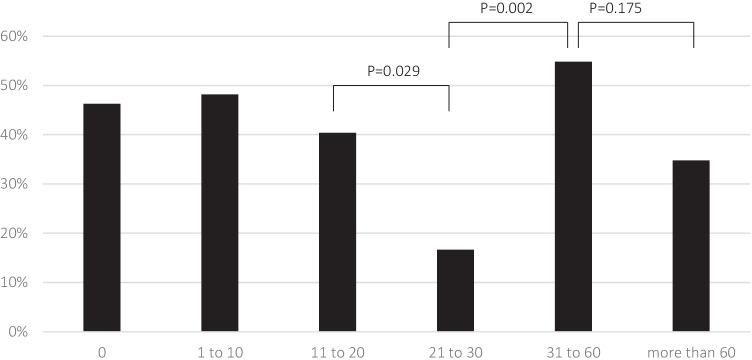
Percentage of patients feeling understood by colleagues and employers subdivided according to the number of days

Figure 1 depicts the proportion of patients feeling understood by employers and colleagues for each subgroup. The percentage was significantly lower in patients with 21 to 30 than 11 to 20 sick days (*P* = 0.029; OR = 0.295, 95% CI 0.097–0.895).

More participants with 31 to 60 sick days felt understood by colleagues and employers than with 21 to 30 days (*P* = 0.002, OR = 6.071, 95% CI 1.842–20.009). However, the difference between the categories of 31 to 60 days and more than 60 days was statistically not significant (*P* = 0.175).

The anxiety subscale of the HADS was completed by 374 participants (38 n.r.). Patients with episodic CH had reached an average score of 8 ± 4 points (median 8 points); 151 (151/286, 52.8%, 13 n.r.) had scored eight or more points. Patients with chronic CH had scored 10 ± 5 points (median 9 points); 57 (57/88, 64.7%, 11 n.r.) had reached eight or more points. Patients feeling understood by their colleagues and employers scored less in the HADS-A (*P* < 0.001).

Three-hundred eighty-two participants completed the depression subscale of the HADS (30 n.r.). Patients with episodic CH had reached an average score of 6 ± 4 points (median 6 points); 107 (107/282, 37.9%, 17 n.r.) had scored eight or more points. Patients with chronic CH had scored 9 ± 5 points (median 9 points); 50 (50/87, 57.5%, 12 n.r.) had reached eight or more points. Patients feeling understood by their colleagues and employers scored less in the HADS-D (*P* < 0.001).

**Fig. 2:**
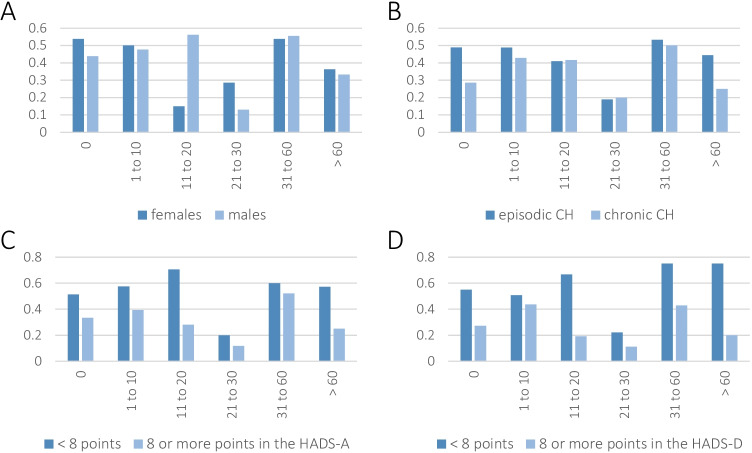
Interaction of sick days and perceived understanding and acceptance by colleagues and employers in different subgroups. The *x*-axis indicates different numbers of sick days; the *y*-axis indicates the proportion of participants feeling understood and accepted. Diagram A compares female (*N* = 132) and male participants (*N* = 279). Diagram B compares patients with episodic (*N* = 299) and chronic (*N* = 99) cluster headache (CH). Diagram C compares patients who scored eight or more points (*N* = 219) in the anxiety subscale of the hospital anxiety and depression scale (HADS) with patients who scored less (*N* = 168). Finally, diagram D compares patients who scored eight or more points (*N* = 162) in the depression subscale of the hospital anxiety and depression scale (HADS) with patients who scored less (*N* = 220)

Figure 2 depicts the same analysis as Fig. 1 but for different subgroups. In particular, it shows a graphical comparison of women and men, and episodic and chronic CH as well as patients scoring less and at least eight points in the anxiety and depression subscales of the HADS. We received information on both the number of missed leisure days in the last 3 months and the understanding from family and friends from 769 participants. Of them, 517 were male (67.2%); the average age was 41 ± 11 years. Episodic CH was reported by and validated in 518 (518/735, 70.5%, 34 n.r.) of whom 416 were in-bout (416/518, 80.3%).

The majority of the participants reported between 0 and 20 days with missed social activities (514/769, 66.9%). Patients with episodic CH had missed 17 ± 21 days (median: 9 days), and patients with chronic CH had missed 30 ± 27 days (median: 21 days); all participants taken together had missed 21 ± 24 days (median: 10 days). Again, we subdivided the participants into subgroups according to their number of missed activities in the last 3 months.

There were 46 patients with zero missed leisure days, 341 with 1 to 10, 127 with 11 to 20, 70 with 21 to 30, 35 with 31 to 40, 48 with 41 to 50, 29 with 51 to 60 and 22 with 61 to 70 missed leisure days. Finally, there were 51 with more than 70 missed leisure days.

Figure 2 depicts the proportion of patients feeling understood by family and friends for each subgroup. The percentage tended to be lower in patients with 31 to 40 missed leisure days than in the group with 21 to 30 days (*P* = 0.079; OR = 0.442, 95% CI 0.189–1.033). There was no statistically significant difference between the 31 to 40-day and 41 to 50-day groups (*P* = 0.372).

## Discussion

**Fig. 3:**
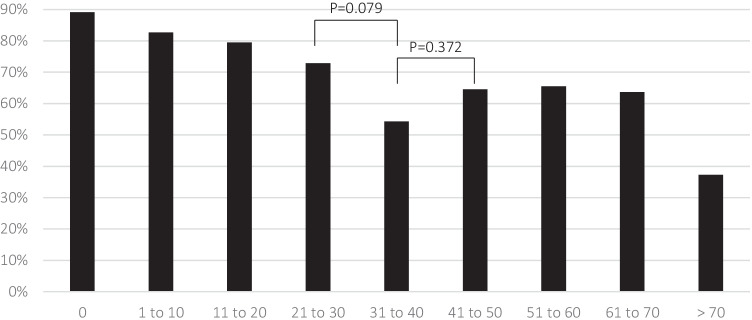
Percentage of patients feeling understood by family and friends subdivided according to the number of days missed social activities in the last three months

We analysed the correlation between feeling understood and both sick days and missed leisure days in patients suffering from CH. The first finding (see Fig. 1 and Fig. 3) is that others’ understanding as felt by the patients declines with increasing absenteeism; we hypothesise that this correlation reflects the frustration that patients perceive in others. However, with a certain number of missed days—between one-third and half of the highest possible numbers, a paradoxical increase in perceived understanding occurs despite patients meeting others’ expectations even less (hence the ‘comprehension paradox’). Thus, frustration caused by CH patients’ absenteeism is unlikely to be the only determinant of colleagues’ comprehension.

One would expect that frequent absences prompt others to reduce their expectations and get used to the patients’ incapacitation. In this case, however, the second drop in understanding with very high numbers of sick days remained unexplained (see the categories with the highest number of sick days in Fig. 1 and Fig. 3).

Flu, back pain and accidents are among the most commonly named reasons for sick leave and, usually, the duration of the absence roughly correlates with the severity of the disease [5]. Sick leave caused by a headache attack is typically short; the absence duration does not reflect the disease burden, which might prevent outsiders from seeing how incapacitating the condition is. However, as absenteeism is the only visible consequence of the disorder, they might struggle to be compassionate.

We hypothesise that compassion will rise despite growing frustration as soon as patients are absent for more extended periods in a row because then the total duration of the absence correlates with the claimed disease severity. A higher number of sick days might make at least some peers understand what it means to bear the disorder. Only when the concerned person meets no expectations anymore due to (almost) permanent incapacitation compassion will drop again.

In the subgroup analyses (see Fig. 2), we found similar drops and subsequent rises in felt understanding. However, the number of sick days after which the drop occurred and the overall experience of others’ comprehension differed. For example, women experienced others’ understanding declines earlier than men did. However, the subsequent rise occurred with a similar number of sick days in both sexes (see Fig. 2A). This finding may imply that the initial drop depends on the individual whereas the subsequent rise does not.

One possible explanation for the differences between women and men might be that women are more prone to presenteeism [2, 9]. Moreover, they often report not wanting to burden colleagues as one reason for presenteeism [15] and, consequently, might be more sensitive to or more often assume others’ decreasing understanding.

Patients with episodic and chronic CH felt understood to a similar extent (see Fig. 2B). This finding may imply that the duration of unremitted disease activity and the precise diagnosis do not influence feeling understood. Instead, it suggests that the number of sick days has greater influence than the precise diagnosis or the duration of unremitted disease activity—as predicted by the above-formulated hypothesis.

When comparing patients with higher anxiety levels with those who scored lower in the HADS-A (see Fig. 2C), we found that the former generally felt less understood, irrespective of their number of sick days. This finding indicates that individual factors may determine the overall estimation of others’ understanding. In particular, several anxious patients might fear others’ negative evaluation in the context of social anxiety [23]. This conclusion might also explain the earlier occurrence of the drop in felt comprehension in anxious persons (see Fig. 2C) and underline that individual factors likely determine the drop and perceived understanding.

Lastly, we also compared patients with higher scores in the depression subscale of the HADS with those who reached lower scores (see Fig. 1D); the findings were similar to those of anxious patients (see Fig. 1C). Likewise, overall felt understanding was lower, and the drop occurred earlier than in patients with less depressive symptoms—possibly due to projection of negative self-evaluation [4] into others, while the rises co-occurred in both groups.

Similar to migraineurs [13], patients generally experienced family members and friends as more understanding than colleagues and employers. Since attacks often occur at night, family members might have witnessed attacks more often than colleagues and hence show greater empathy. Moreover, affection for each other is likely greater among family members than among colleagues. Besides, tasks and expectancies at the workplace are probably more precisely formulated, and deviation becomes obvious more quickly; hence, criticisms and ill feelings might arise more rapidly.

In our analyses (see Fig. 1 and Fig. 3), understanding first declined and later increased numerically with an increasing number of missed days; however, only among colleagues and employers, comprehension increased statistically significantly. We believe that the number of colleagues with whom one works regularly might be smaller than the number of family members and friends. Besides, the former might all receive similar information, while the latter’s state of knowledge might diverge more.

Many patients with cluster headache [17] and some patients with migraine [13] do not feel understood by their colleagues and employers. The subgroup analyses (see Fig. 2) indicate that various factors foster or prevent the appearance of that feeling, many of which seem to depend on patients themselves. However, it suggests itself that others also play a major role, namely through emotional validation—or the lack thereof.

The term emotional validation refers to communicating the acceptance and understanding of another person’s suffering. Research by Edlund et al. indicates that spouses trained and encouraged to validate their partner’s feelings strongly reduce the patient’s negative emotions [6]. Consistently, our previous research confirms the importance of CH patients’ relationships on their well-being [18].

The self-verification theory provides a broader theoretical framework to appreciate the importance of others’ understanding [22]. According to this theory, individuals seek reactions from others that support their self-view and help verify one’s own experiences. Ultimately, this process contributes to emotional self-regulation and prevents being emotionally overwhelmed [1].

Thus, irrespective of the patients themselves, their CH attacks and their comorbidities, their social circles significantly affect their emotional state. Interestingly, feeling understood is associated with increased well-being [14]. Accordingly, in our sample, participants feeling understood by their colleagues scored lower on the anxiety and depression subscales of the HADS.

Consequently, education of patients’ significant others might prevent the comprehension paradox. In other words, a holistic therapeutic approach taking into account not just the patients but also their families might help patients feel understood even when the number of sick days or missed leisure days does not make their suffering evident to others, yet.

### Limitations

This study does not provide evidence about the reasons for the comprehension paradox. Moreover, we assessed whether patients *felt* that others understand them—but not if others *did* understand them. Therefore, we would like to encourage further research on the perspectives of headache patients’ social circles; in future studies, colleagues, employers, friends and family members should be inquired directly.

Previous studies did not investigate the correlation between absenteeism and understanding but the absence from work in general. The average number of sick days was higher in our sample than in the previously published samples [7, 8, 12, 16], likely indicating that our participants were more severely affected [3]. However, participation bias should not influence our conclusions, as they do not require a representative sample. Nevertheless, we encourage confirmatory studies.

A further limitation is that the highest possible number of sick days and missed leisure days is not specified precisely in the Headache-Attributed Lost Time (HALT) Indices [19] used in this study. Given that both diagrams had comparable patterns (see Fig. 1 and Fig. 3), we do not think that a measuring bias compromised our conclusions. Nevertheless, future studies could benefit from a more precise definition. Moreover, the number of sick days was self-reported; the data therefore likely comprises a recall bias. We discussed further limitations associated with the study design in a previous paper [3]. Most importantly, the validation of the headache diagnosis relied on self-reported symptoms, not on a validated questionnaire, which might have introduced a sampling error.

## Conclusion

The number of CH patients feeling understood by others decreases with an increasing number of sick days. Their social circles’ frustration with the patients’ failure to meet obligations and expectations are a likely reason. With a growing number of sick days, however, the portion of patients feeling understood rises again. This ‘comprehension paradox’ implies the influence of other factors. We suspect that growing numbers of sick days foster understanding as the disability of the disease becomes increasingly apparent.

## Data Availability

The datasets used and analysed during the current study are available from the corresponding author on reasonable request.

## Abbreviations

CH: Cluster headache
CI: Confidence intervals
HADS: Hospital Anxiety and Depression Scale
HALT: Headache-attributed lost time
n.r.: Not reported
OR: Odds ratios
